# Supplementary material for: Phosphoramidate Azole Oligonucleotides for Single Nucleotide Polymorphism Detection by PCR
Source: Int J Mol Sci. 2024 Jan 3;25(1):617. doi: 10.3390/ijms25010617 (PMC10778797; doi:10.3390/ijms25010617)
Supplement: Supplementary file 1 [file ijms-25-00617-s001.zip › ijms-2777413-supplementary.pdf]

Supplementary materials

Phosphoramidate Azole Oligonucleotides for Single Nucleo-tide Polymorphism Detection by PCR

Alexey S. Chubarov, Elizaveta E. Baranovskaya, Igor P. Oscorbin, Ivan I. Yushin, Maxim L. Filipenko, Dmitrii V. Pyshnyi, Svetlana V. Vasilyeva and Alexander A. Lomzov

Institute of Chemical Biology and Fundamental Medicine, SB RAS, 8 Lavrentiev Avenue, Novosibirsk 630090, Russia

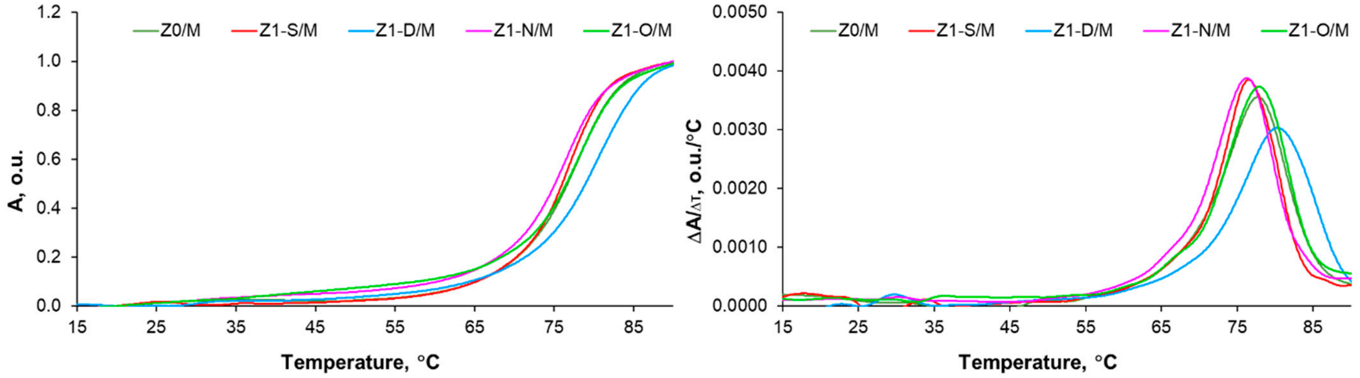

Figure S1. Normalized UV-melting curves (left) and differential of UV-melting curves (right) for Z/T-series. The condition mimics the PCR buffer (6 mM $MgCl_2$, 75 mM NaCl, 10 mM sodium cacodylate buffer, pH 7.2). The oligonucleotide concentration is 2.5 μM.

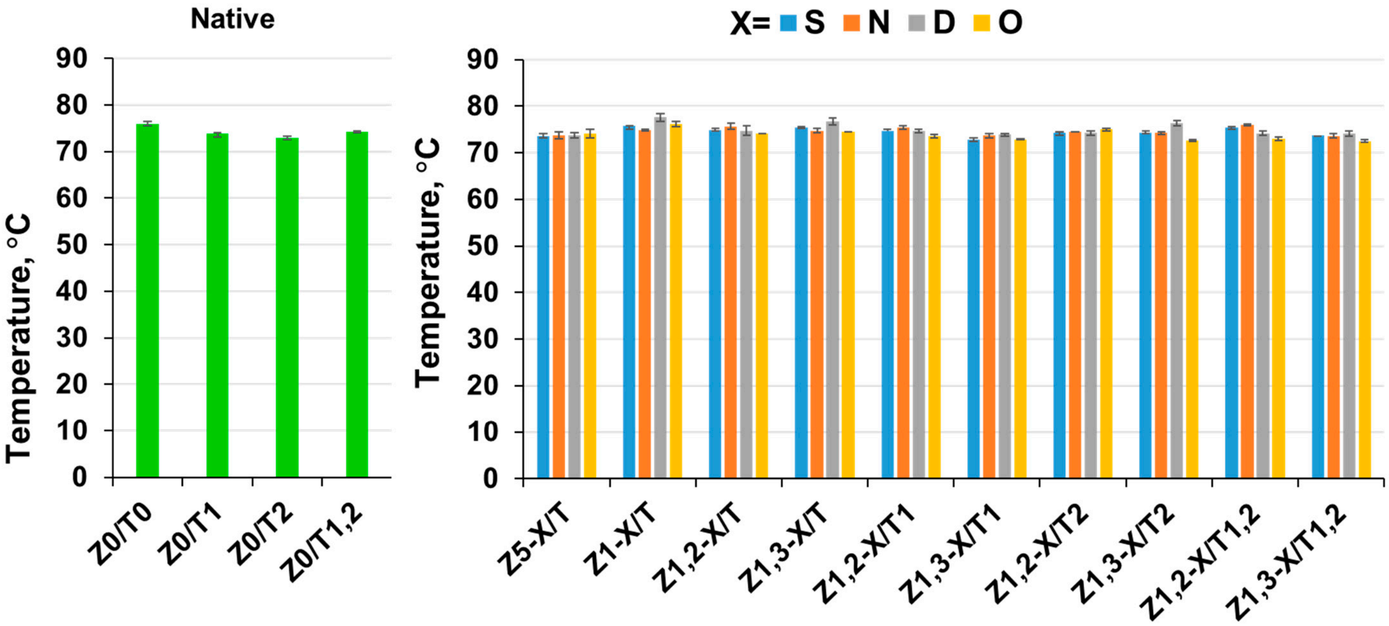

Figure S2. Melting temperature values of Z/T-series were obtained via UV-melting curve fitting using the two-state model. The condition mimics the PCR buffer (6 mM $MgCl_2$, 75 mM NaCl, 10 mM sodium cacodylate buffer, pH 7.2). The oligonucleotide concentration is 2.5 μM.

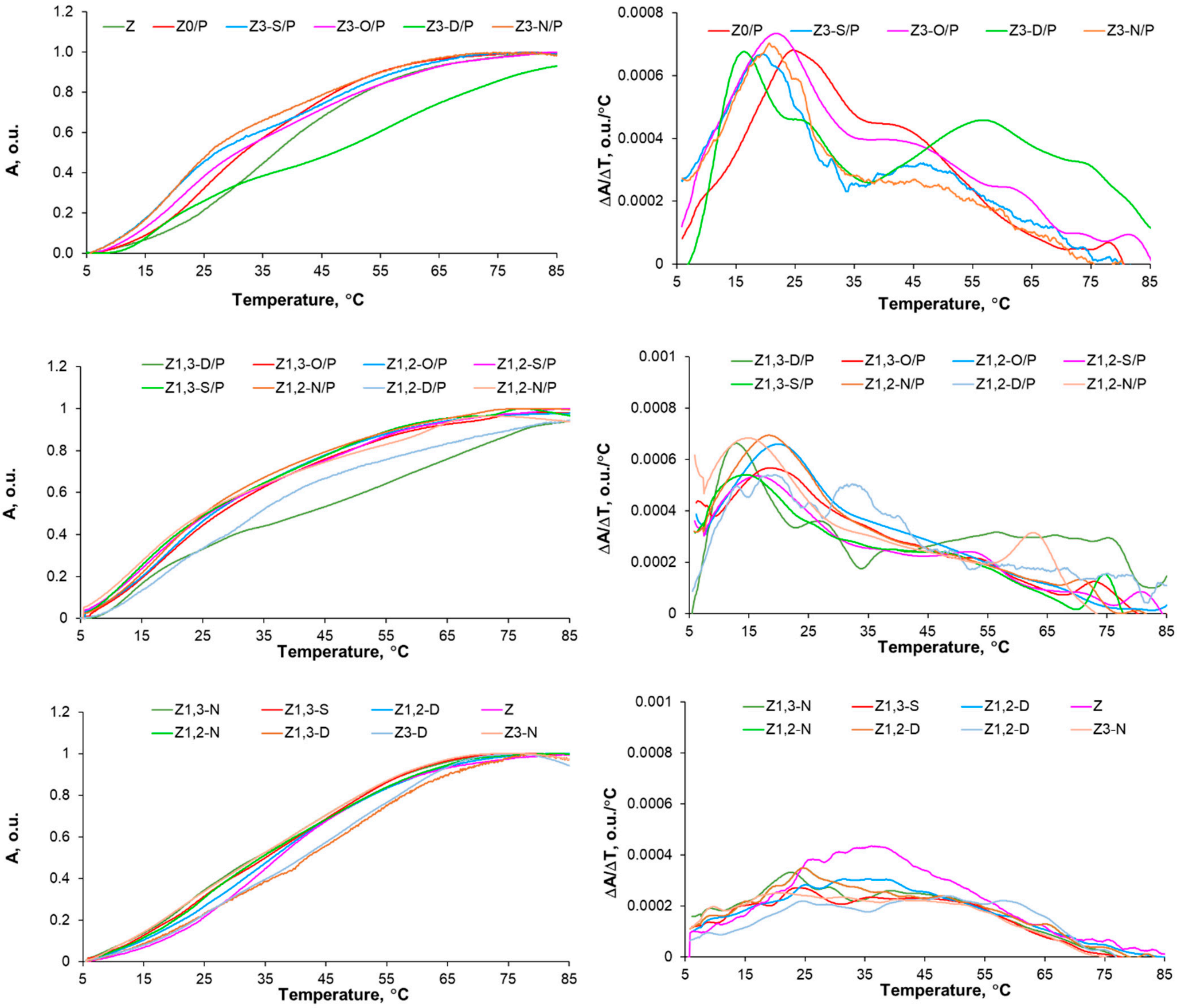

Figure S3. Normalized UV-melting curves (left) and differential of UV-melting curves (right) for Z/P-series (top, middle) and Z oligonucleotides (bottom). The condition mimics the PCR buffer (6 mM $MgCl_2$, 75 mM NaCl, 10 mM sodium cacodylate buffer, pH 7.2). The oligonucleotide concentration is 2.5 μM.

Table S1. Thermodynamic parameters of the Z/T-series complexes obtained via UV-melting curve fitting using the two-state model. The condition mimics the PCR buffer (6 mM $MgCl_2$, 75 mM NaCl, 10 mM sodium cacodylate buffer, pH 7.2). The oligonucleotide concentration is 2.5 μM.

Complex	ΔH°, kcal/mol	ΔS°, cal/(mol*K)	$ΔG°_{37}$, kcal/mol	T_m, °C
Z0/T0	-130 ± 5	-345 ± 15	-22.9 ± 0.6	75.9 ± 0.4
Z0/T1	-126 ± 11	-337 ± 33	-21.8 ± 1	73.9 ± 0.7
Z0/T2	-140 ± 5	-378 ± 15	-22.9 ± 0.5	72.9 ± 0.2
Z0/T1,2	-127 ± 11	-339 ± 32	-22 ± 1.2	74.2 ± 0.1
Z1-S/T	-143 ± 21	-384 ± 60	-24.3 ± 2.1	75.7 ± 0.5
Z5-S/T	-154 ± 22	-416 ± 64	-24.6 ± 2.5	73.6 ± 0.4
Z1,2-S/T	-120 ± 7	-317 ± 20	-21.4 ± 0.7	74.9 ± 0.2
Z1,3-S/T	-115 ± 9	-302 ± 26	-21 ± 0.9	75.3 ± 0.4
Z1,2-S/T-1	-141 ± 18	-379 ± 53	-23.7 ± 1.9	74.7 ± 0.2
Z1,3-S/T-1	-136 ± 11	-366 ± 31	-22.5 ± 1	72.9 ± 0.3
Z1,2-S/T-2	-135 ± 6	-361 ± 19	-22.9 ± 0.6	74.4 ± 0.3
Z1,3-S/T-2	-132 ± 14	-354 ± 41	-22.9 ± 0.9	74.2 ± 0.2
Z1,2-S/T-1,2	-136 ± 11	-362 ± 32	-23.3 ± 1.1	75.4 ± 0.5
Z1,3-S/T-1,2	-142 ± 8	-382 ± 22	-23.4 ± 0.8	73.8 ± 0.2
Z1-N/T	-135 ± 16	-362 ± 46	-23.1 ± 1.7	74.9 ± 0.1
Z5-N/T	-128 ± 5	-343 ± 16	-22 ± 0.3	73.8 ± 0.8
Z1,2-N/T	-124 ± 6	-328 ± 18	-22.1 ± 0.5	75.7 ± 0.6
Z1,3-N/T	-113 ± 3	-297 ± 8	-20.6 ± 0.2	74.7 ± 0.4
Z1,2-N/T-1	-135 ± 5	-359 ± 15	-23.2 ± 0.5	75.4 ± 0.3
Z1,3-N/T-1	-139 ± 10	-373 ± 30	-23.1 ± 0.9	73.7 ± 0.4
Z1,2-N/T-2	-145 ± 13	-390 ± 37	-24 ± 1.4	74.5 ± 0
Z1,3-N/T-2	-140 ± 15	-376 ± 45	-23.4 ± 1.6	74.3 ± 0.3
Z1,2-N/T-1,2	-127 ± 6	-337 ± 16	-22.5 ± 0.6	75.9 ± 0.2
Z1,3-N/T-1,2	-140 ± 19	-376 ± 55	-23.1 ± 1.8	73.6 ± 0.5
Z1-D/T	-125 ± 9	-331 ± 26	-22.9 ± 0.8	77.6 ± 0.8
Z5-D/T	-127 ± 7	-340 ± 21	-21.8 ± 0.6	73.7 ± 0.6
Z1,2-D/T	-112 ± 7	-296 ± 18	-20.6 ± 1	74.7 ± 1
Z1,3-D/T	-105 ± 5	-273 ± 15	-20.3 ± 0.4	76.7 ± 0.7
Z1,2-D/T-1	-131 ± 9	-348 ± 27	-22.5 ± 0.9	74.7 ± 0.4
Z1,3-D/T-1	-132 ± 14	-352 ± 42	-22.4 ± 1.5	73.9 ± 0.2
Z1,2-D/T-2	-132 ± 11	-352 ± 31	-22.5 ± 1	74.2 ± 0.4
Z1,3-D/T-2	-129 ± 13	-343 ± 38	-22.9 ± 1.3	76.3 ± 0.6
Z1,2-D/T-1,2	-129 ± 15	-344 ± 44	-22.2 ± 1.5	74.2 ± 0.5
Z1,3(D)/T-1,2	-121 ± 7	-323 ± 22	-21.4 ± 0.6	74.1 ± 0.6
Z1-O/T	-135 ± 11	-359 ± 31	-23.5 ± 1	76.1 ± 0.6
Z5-O/T	-130 ± 9	-347 ± 25	-22.3 ± 0.6	74.2 ± 0.9
Z1,2-O/T	-140 ± 0	-376 ± 1	-23.3 ± 0	74.1 ± 0
Z1,3-O/T	-125 ± 2	-334 ± 5	-21.9 ± 0.2	74.4 ± 0
Z1,2-O/T-1	-135 ± 13	-361 ± 38	-22.6 ± 1.3	73.6 ± 0.3
Z1,3-O/T-1	-136 ± 8	-367 ± 22	-22.5 ± 0.7	72.9 ± 0.1
Z1,2-O/T-2	-136 ± 13	-363 ± 38	-23.2 ± 1.4	75 ± 0.2
Z1,3-O/T-2	-132 ± 4	-355 ± 12	-22 ± 0.4	72.6 ± 0.2
Z1,2-O/T-1,2	-135 ± 15	-362 ± 44	-22.4 ± 1.4	73.1 ± 0.4
Z1,3-O/T-1,2	-143 ± 9	-388 ± 27	-23.1 ± 0.9	72.5 ± 0.3

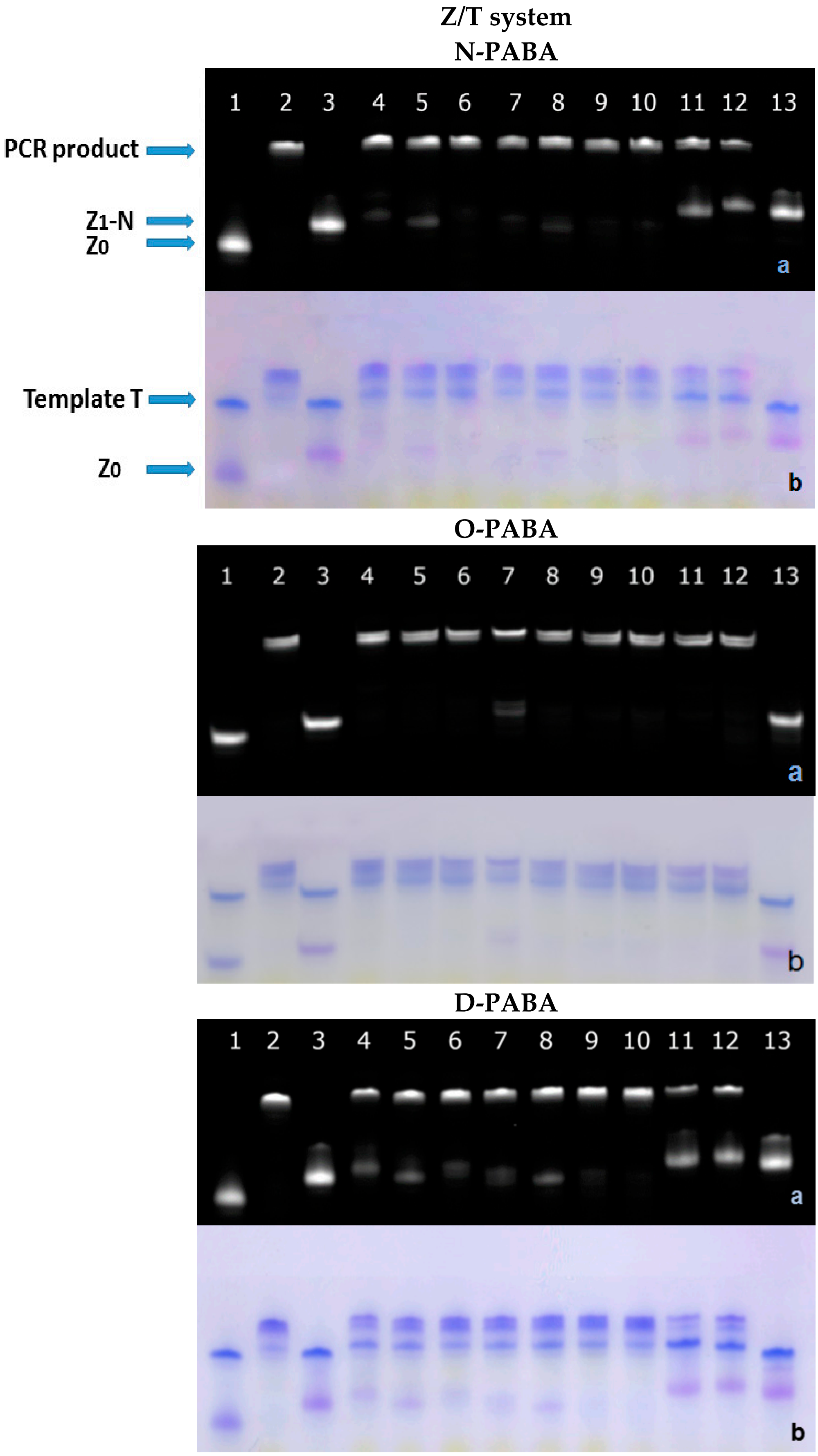

Figure S4. Gel electrophoresis images of elongation products for Z/T system in FAM channel (a) and StainAll staining (b). Primer/template mixture controls: lane 1 Z0/T, lane 3 Z1/T, and lane 13 Z1,2/T. Mixtures after PCR: lane 2 Z0/T, lane 4 Z1/T, lane 5 Z2/T, lane 6 Z3/T, lane 7 Z4/T, lane 8 Z5/T, lane 9 Z6/T, lane 10 Z7/T, lane 11 Z1,2/T, and lane 12 Z1,3/T.

Z/T-1 system

N-PABA

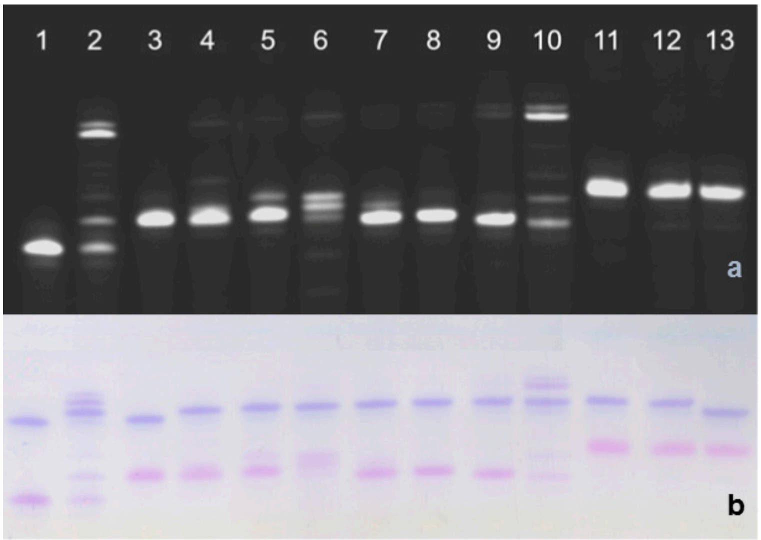

O-PABA

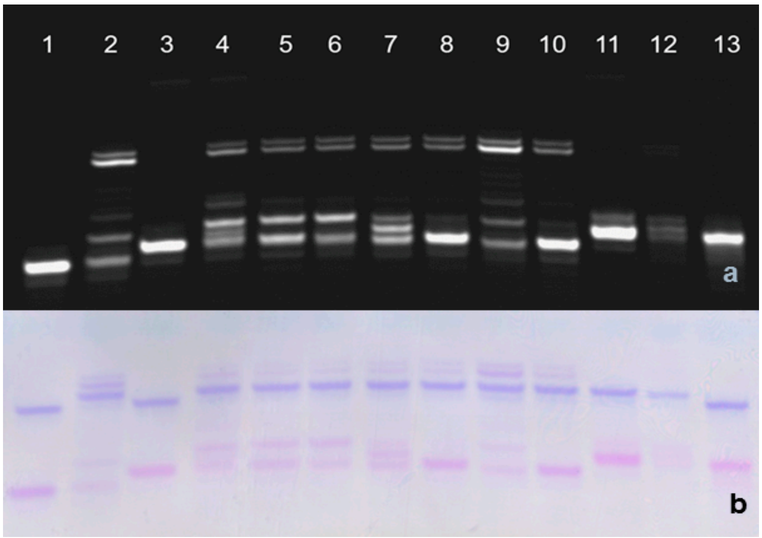

D-PABA

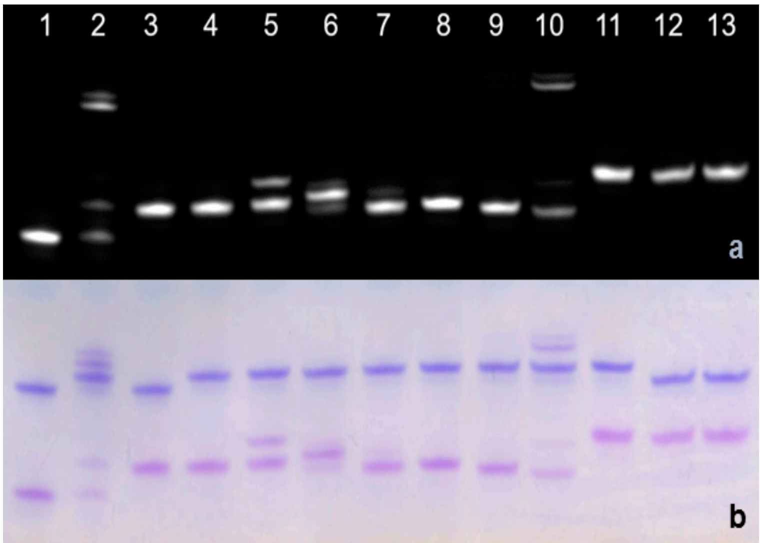

Figure S5. Gel electrophoresis images of elongation products for Z/T-1 system in FAM channel (a) and StainAll staining (b). Primer/template mixture controls: lane 1 Z0/T-1, lane 3 Z1/T-1, and lane 13 Z1,2/T-1. Mixtures after PCR: lane 2 Z0/T-1, lane 4 Z1/T-1, lane 5 Z2/T-1, lane 6 Z3/T-1, lane 7 Z4/T-1, lane 8 Z5/T-1, lane 9 Z6/T-1, lane 10 Z7/T-1, lane 11 Z1,2/T-1, and lane 12 Z1,3/T-1.

Z/T-2 system

N-PABA

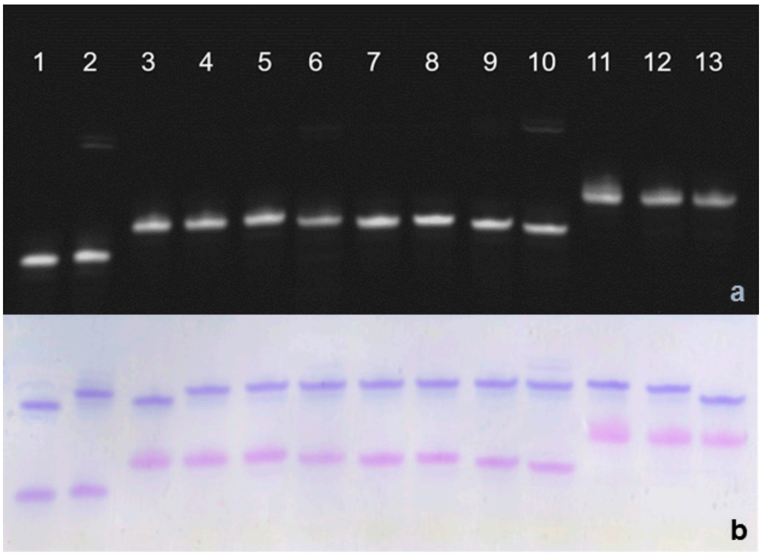

O-PABA

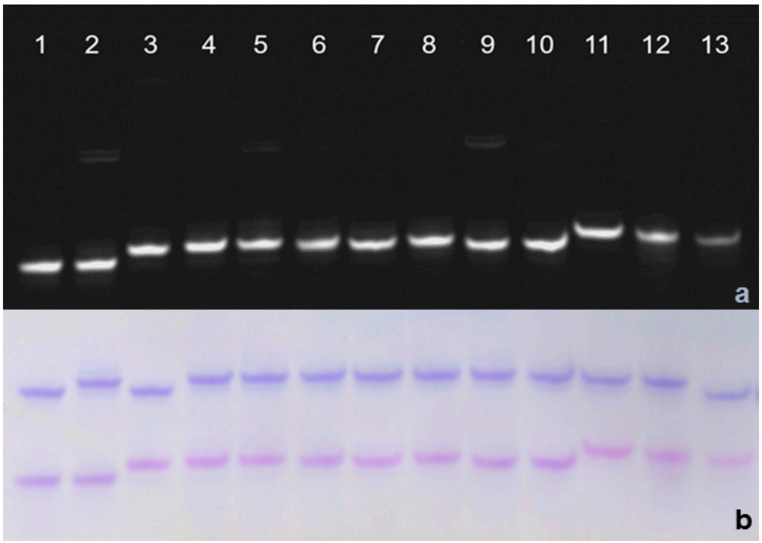

D-PABA

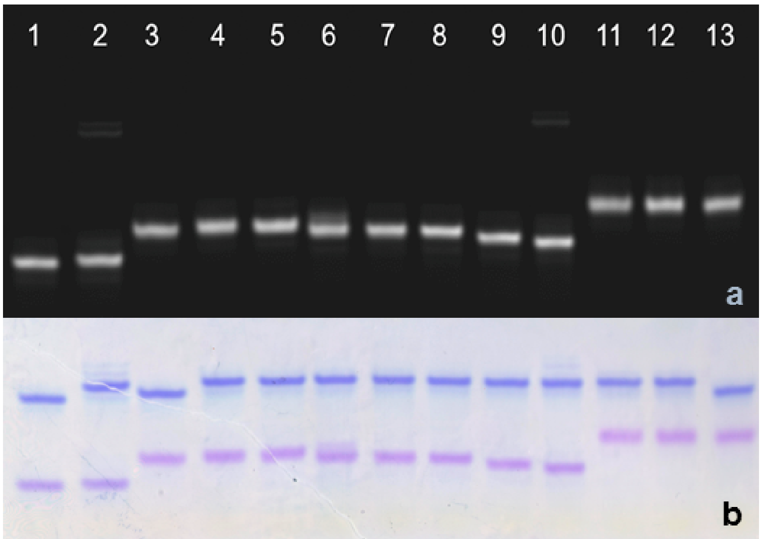

Figure S6. Gel electrophoresis images of elongation products for Z/T-2 system in FAM channel (a) and StainAll staining (b). Primer/template mixture controls: lane 1 Z0/T-2, lane 3 Z1/T-2, and lane 13 Z1,2/T-2. Mixtures after PCR: lane 2 Z0/T-2, lane 4 Z1/T-2, lane 5 Z2/T-2, lane 6 Z3/T-2, lane 7 Z4/T-2, lane 8 Z5/T-2, lane 9 Z6/T-2, lane 10 Z7/T-2, lane 11 Z1,2/T-2, and lane 12 Z1,3/T-2.

Z/T-1,2 system

N-PABA

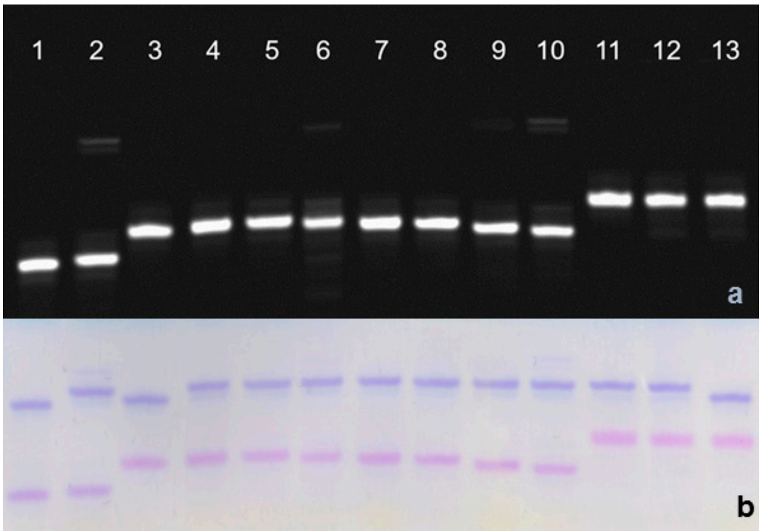

O-PABA

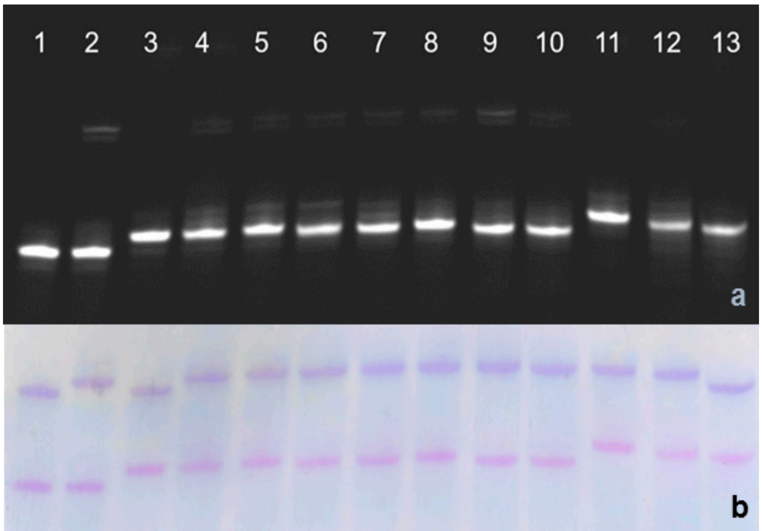

D-PABA

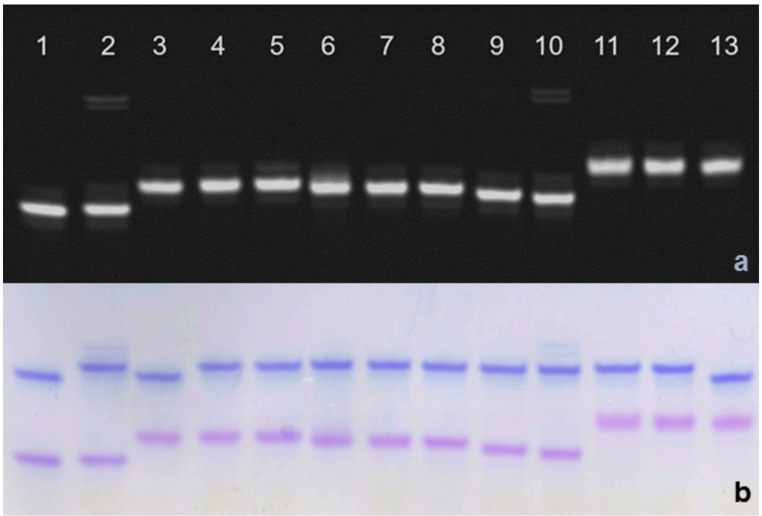

Figure S7. Gel electrophoresis images of elongation products for Z/T-1,2 system in FAM channel (a) and StainAll staining (b). Primer/template mixture controls: lane 1 Z0/T-1, lane 3 Z1/T-1,2, and lane 13 Z1,2/T-1,2. Mixtures after PCR: lane 2 Z0/T-1,2, lane 4 Z1/T-1,2, lane 5 Z2/T-1,2, lane 6 Z3/T-1,2, lane 7 Z4/T-1,2, lane 8 Z5/T-1,2, lane 9 Z6/T-1,2, lane 10 Z7/T-1,2, lane 11 Z1,2/T-1,2, and lane 12 Z1,3/T-1,2.

Z/P system

N-PABA

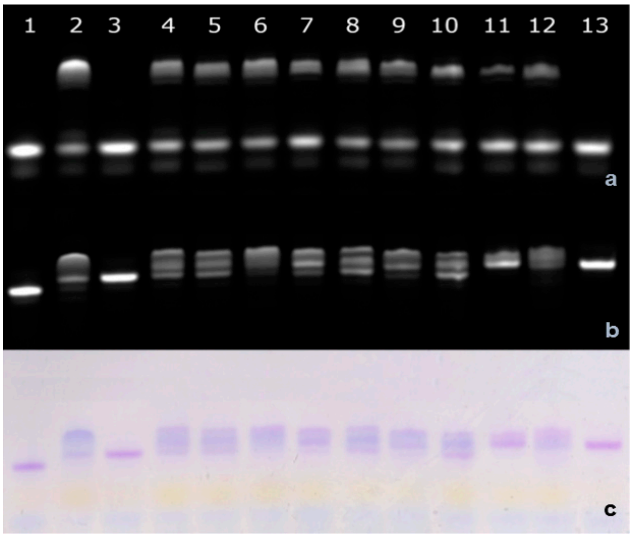

O-PABA

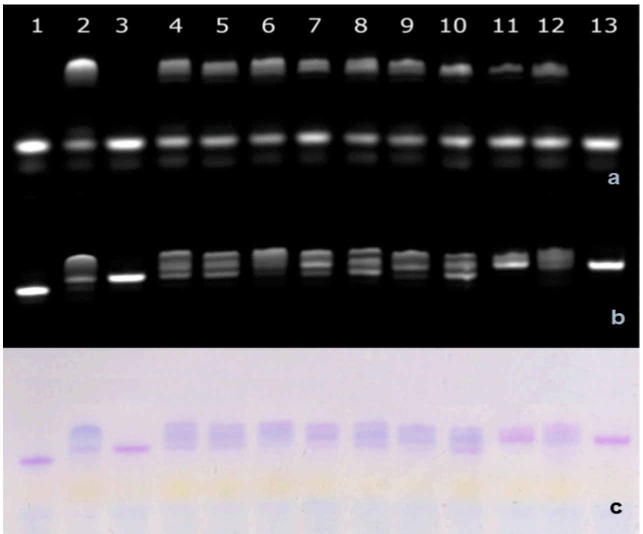

D-PABA

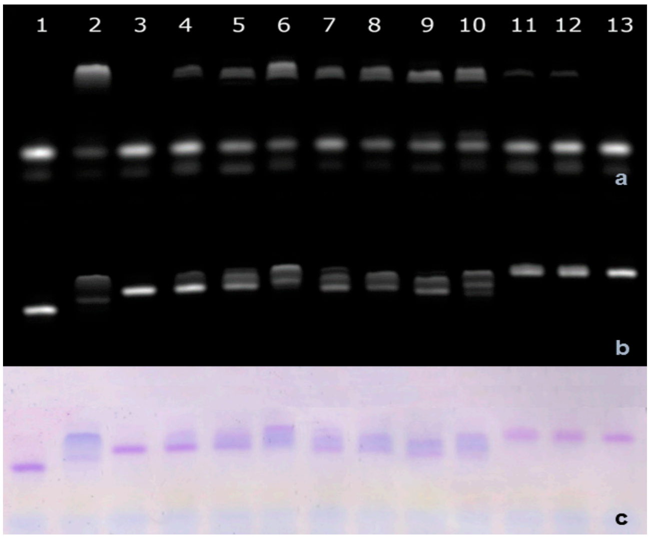

Figure S8. Gel electrophoresis images of PCR product for Z/P system in Cy5 channel (a), FAM channel (b), and StainAll staining (c). Primer/template mixture controls: lane 1 Z0/P, lane 3 Z1/P, and lane 13 Z1,2/P. Mixtures after PCR: lane 2 Z0/P, lane 4 Z1/P, lane 5 Z2/P, lane 6 Z3/P, lane 7 Z4/P, lane 8 Z5/P, lane 9 Z6/P, lane 10 Z7/P, lane 11 Z1,2/P, and lane 12 Z1,3/P.
